# Supplementary material for: Clinical outcomes of adults hospitalized for laboratory confirmed respiratory syncytial virus or influenza virus infection
Source: PLoS One. 2021 Jul 22;16(7):e0253161. doi: 10.1371/journal.pone.0253161 (PMC8297903; doi:10.1371/journal.pone.0253161)
Supplement: Attachment — Submitted filename: Response_to_Reviewers.docx [file pone.0253161.s002.docx]

PONE-D-21-03134
Clinical outcomes of adults hospitalized for laboratory confirmed respiratory syncytial virus or influenza virus infection .
PLOS ONE

**Rebuttal**

We would like to thank all reviewers for their time and scrutiny to improve this manuscript. We hope that our responses will be satisfying. Please find below our point-by-point reply:

**Reviewer #1:**

This is an interesting piece of research that has not been explored well enough. However, I have a few questions and it relates mainly toward the small number of events and the analysis.

1. The outcomes table should be placed in the main manuscript instead of the supplementary material.

**Response:** This is an excellent suggestion, and we would like to thank you for that. We moved the outcomes table in the main manuscript.

2. I’m trying to understand how the data has been collected as the study design section mentioned “retrospective analysis” and study population and data collection section writes “prospectively collected data”. Were these patients prospectively recruited or were cases identified through the hospital surveillance program? If the latter, would it have been possible to increase the sample size to another season as the number of events of the RSV groups is very low (n=15).

**Response:** Thank you for raising this question which gives us the opportunity to provide some clarifications. The study population was indeed identified through the hospital surveillance program. Since the hospital’s microbiology laboratory introduced combined PCR testing for RSV and influenza only at the beginning of October 2017, these are all available data. Based on your comment that it is not entirely clear how the data were collected, we have taken the liberty of revising the methodology section and hope that it is more understandable now.

The section now reads as follows (page 4, lines 82-102 and page 5, lines 103-113):

**Study design**

We performed a retrospective analysis from patients with laboratory confirmed influenza or RSV infection, who were admitted to one of the two acute care hospitals in the canton Thurgau during winter season I (2017/2018) or II (2018/2019).

**Study setting and population**

The Thurgau hospital group has about 570 acute care beds. To prevent the spread of influenza in the hospital, the division of infection control established a new surveillance program in October 2017 for patients who are being hospitalized with influenza or RSV infection. At this time, the microbiology laboratory introduced a combined RT-PCR for the diagnosis of influenza and RSV infection. Clinicians were advised to obtain a nasopharyngeal swab for microbial diagnosis of influenza or RSV as a standard diagnostic procedure in all patients with symptoms and signs of acute respiratory infection (ARI) and whose’ condition required hospital admission. Patients with ARI presented with an influenza-like illness consisting of fever, general malaise, cough, or myalgia or had a chest infiltrate suggestive for viral pneumonia. Patients hospitalized on the wards who developed new ARI also underwent testing for influenza and RSV. All patients who tested positive for influenza were immediately placed in droplet isolation as per local infection control guideline.

**Data collection**

The hospital influenza and RSV surveillance served as basis for our study. As part of this surveillance program, the division of infection control routinely collects data on microbiological diagnosis, length of hospital stay, admission to ICU, and death during hospital stay in patients who test positive for influenza virus or RSV by means of an RT-PCR. This prospective surveillance starts in week 44 in the previous year and extends to week 16 in the following year.

We included all adult patients (equal or older than 18 years of age at time of hospitalization) who were hospitalized during influenza seasons I and II with a laboratory confirmed influenza or RSV infection. Only the first episode was considered. We excluded patients with an RSV/influenza co-infection. Additional data on patient demographics, comorbidities, vital signs on admission, laboratory results, treatment and outcomes were retrospectively retrieved from individual electronic patient files. These data were entered into the electronic patient file during routine patient care. Every death in the community is automatically reported to the public hospitals in the canton and is therefore visible in the electronic health record. This enabled us to also determine the 30-day mortality.

3. Can the authors elaborate on how the cases with ARI were identified? Were they based on ED diagnosis codes? In addition, I see that all 588 patients had a chest radiograph. Is that a requirement for all ARI admissions?

**Response:** We are grateful for this question. Before introducing the combined PCR, clinicians were instructed about the clinical and radiological criteria they should apply to decide whether a patient qualifies for a nasopharyngeal swab. However, whether a patient was tested by PCR eventually remained at the discretion of the responsible physicians working in the ED. The majority of patients with new clinical signs of ARI receive at least a chest X-ray. In this cohort, 454 of 548 (82.8%) received a chest x-ray. Since the question relates to the previous one, we kindly ask you to review the revisions already mentioned above under point 2. We hope that our revision will sufficiently answer your question.

4. The follow-up time here is only 7 days and likely to encounter ties in their analysis. Can the authors explain the rationale in performing a PH model instead of a simpler logistic regression model?

**Response:** We would like to thank the reviewer for this important question that enables us to improve the statistical analyzes and draw correct inferences. We agree that we would need to consider ties in our Cox PH regression model. This could be done by an Efron or Breslow approximation. However, instead of performing a more complex analysis that would be less understandable we decided to redo our main analysis by applying simple binary logistic regression. With the new analysis, main outcomes and inferences that can be drawn remain the same. We kindly ask you to refer to our response under the point 5 to review all changes that have been made to the manuscript.

5. The authors adjusted for age and comorbidities. In my opinion, that might not be necessary since they were found to be insignificant in Table 1. Why was adjustment only done for pulmonary infiltrate but not the other clinical features found significant on admission in Table 1?

**Response:** Again, we are grateful for raising this important issue. Being in line with your previous suggestion to rather stick with simple logistic regression, we repeated our multivariable analysis and entered only those predictors that were statistically significant in univariate analyses. This procedure also enabled us to be parsimonious with the predictors entered and eventually prevented from overfitting of the model.

The concerning statistics and results sections have been revised accordingly:

Page 7, lines 138-143: We used binary logistic regression to model the crude prediction of RSV and influenza virus infection for the probability of ICU admission or death within the first 7 days after hospital admission. We then applied multivariable models to adjust for additional predictors of our primary outcome in patients with RSV compared to influenza virus infection. Only predictors that were statistically significant in univariate analyses were considered in the multivariable model.

Page 10, lines 192-199: In the crude analysis, RSV patients were twice as likely to be admitted to ICU or die within 7 days after admission compared to patients with influenza (OR 2.06, 95% CI 1.09-3.90, p=.027) (Table 3). However, the association was no longer significant after controlling for temperature, leukocyte count, and presence of a pulmonary infiltrate on admission. While neither fever nor the underlying respiratory virus appeared to have a significant impact on the primary outcome in the adjusted analysis, a higher leukocyte count (adjusted OR 1.07, 95% CI 1.02-1.13) and the presence of a pulmonary infiltrate on chest X-ray (aOR 3.41, 95% CI 1.93-6.02, p<.001) appeared to significantly increase the risk of an unfavourable outcome.

6. Presence of pulmonary infiltrate has been found to increase the risk of ICU admission or mortality. This was presented as a significant finding and discussed briefly. However, this finding does not seem to align with the objectives of the study of comparing disease severity of RSV vs influenza.
   In the discussion, the authors “suspect the influence of pneumonic infiltrate was so strong that age and comorbidities were no longer relevant..”. From Table 2, it looks like the effects of pneumonic infiltrate attenuating the effect of the RSV instead of these potential confounders. The authors could relate these findings better by further investigating the proportion of patients who experienced the event and had pulmonary infiltrates, stratified by RSV/influenza.

**Response:** We agree with the comment that the finding does not align with the study objectives and thank the reviewer for suggesting an additional analysis. We now performed a subgroup analysis, evaluating the impact of age on ICU admission or death within 7 days after admission in patients presenting with a pulmonary infiltrate stratified by influenza and RSV. A total of 29 (25.2%) influenza cases and 7 (21.9%) RSV cases in this subgroup experienced the primary outcome. The difference was not statistically significant. However, the proportion of cases reaching the outcome between the influenza and RSV group clearly differed by age category with a higher proportion of elderly (over 80 years of age) patients in the RSV group. The numbers were too small to perform any meaningful statistics. We suggest adding this figure to the results and remove the original figure 2 as suggested by reviewer #2.

The revised section reads accordingly:

Page 10, lines 199-202: Patients with pneumonia on admission were 3.41 times more likely to be transferred to ICU or to die within the first 7 days than patients without pneumonia (aOR 3.41, 95% CI 1.93-6.02, p<.001). The addition of this predictor to the model, however, seemed to attenuate the effect of RSV as indicated by a decrease in the adjusted OR.

Page 11, lines 208-216: To better understand the association of a pulmonary infiltrate with clinical outcome, we performed a subgroup analysis, examining the impact of age on ICU admission or death within 7 days after admission in patients presenting with a pulmonary infiltrate stratified by influenza and RSV. First, we found that 29 of 115 (25.2%) influenza cases and 7 of 32 (21.9%) RSV cases in this subgroup experienced the primary outcome. The difference was not statistically significant. However, we note that the proportion of cases reaching the outcome between the influenza and RSV group clearly differed by age category with a higher proportion of elderly (over 80 years of age) patients in the RSV group (Fig 2). The identified virus modified the effect of age on the development of the outcome of ICU admission or death among those with a pulmonary infiltrate.

ICU admisison or death

|  | Influenza | RSV |
| --- | --- | --- |
| 35-65 years | 34.6% | 11.1% |
| 66-80 years | 31.6% | 16.7% |
| > 80 years | 15.7% | 29.4% |

**Reviewer #2: Minor Comments:**

Methods:

- The influence of seasonality (season I vs. season II) on the outcome of the patients is not mentioned. It would be interesting to explore if the RSV/Influenza-associated hospitalizations varied substantially during the study period, related to the particular virus types and subtypes in circulation

**Response:** According to your suggestion we evaluated the influence of seasonality. There were significant differences in the proportion of admissions comparing the two respiratory seasons. We have added the numbers of admission for RSV and influenza separated for the 2 respiratory seasons in Table 1. The percentages of patients in the RSV- and influenza-group experiencing the outcome were surprisingly constant over the two seasons. We suggest adding these results to table 2.

| **ICU or death within 7 days according to season** | **RSV** | | **Influenza** | | **p-value ^a^** |
| --- | --- | --- | --- | --- | --- |
| Admitted in season I, n of total (%) | 5/27 | (18.5) | 25/270 | (9.3) | 0.128 |
| Admitted in season II, n of total (%) | 10/52 | (19.2) | 23/199 | (11.6) | 0.145 |

^a^ p-values were calculated using Chi-square for comparison of proportions.

Unfortunately, we did not collect the data on influenza subtypes. We therefore could not perform such additional analysis which would have been beyond the scope of this manuscript.

Results:

- The authors stated that the proportion of patients with infiltrate on chest X-ray was significantly higher in the RSV group (46.4% vs. 29.9%). However, figure 1 shows 40.5% vs.24.5%. Please revise

**Response:** We thank Reviewer #2 for detecting this oversight. The percentages have been corrected in the abstract and in Table 1.

- Primary endpoint is part of the main objective of this paper but the results are not shown in main body. Please move Suppl. Table 1. to main body

**Response:** This is an excellent suggestion, and we would like to thank you and Reviewer #1 for pointing this out. We moved the outcomes table in the main manuscript as Table 2.

- Justify why the covariates tested in the multivariate model were selected (e.g. Significance criteria/supported by previous literature)

**Response:** we would like to thank you and Reviewer #1 for highlighting this issue. We repeated the complete analysis. We kindly ask you to refer to our response above provided to Reviewer #1 under point 5.

**Reviewer #3:**

**1.** As the inpatient follow up was limited to 7 days, would the authors be expected to fully describe the clinical outcome of adults patients hospitalised with RSV or influenza infection? This is important as "outcomes" is present in both the full and short title of the manuscript.

**Response:** This is an excellent suggestion, and we would like to thank you and the other reviewers for pointing this out. We moved the outcomes table in the main manuscript as Table 2.

**2.** Is Figure 2 necessary? In its current form, it appears to add little to the manuscript as the cutoff is at 7 days

**Response:** Again, this is an excellent remark. We removed this figure. However, since Reviewer #1 recommended performing a subgroup analysis, we propose to add this figure from the subgroup analysis evaluating age as a predictor of the primary outcome in the subgroup of those with a pulmonary infiltrate stratified by the underlying viral disease.

**3.** Please review reference 16 - this research article had described workflow parameters and in its results, it did not describe sensitivity or specificity of the test referred to.

**Response:** We thank the reviewer for pointing out this oversight. The correct reference (Zou Xiaohui et al. Int J Infect Dis. 2019;80:92-7.) which is now reference 18. and two additional references (16. Cohen et al. J Clin Microbiol. 2018;56(2) and 17. Ling et al. J Clin Microbiol. 2018;56(3)) which are related to the validation of this test have been added to the manuscript.

**4.** Would the authors consider expanding their discussion to other points including clinical prediction models for RSV, possible preventive measures, nosocomial transmission, possible planned clinical interventions for RSV.

**Response:** We agree with reviewer #3 that the importance of preventive measures and clinical interventions to prevent nosocomial transmission of RSV cannot be emphasized enough. We have added a statement on the importance of virological diagnosis and prevention.
