# Supplementary material for: Clinical outcomes of adults hospitalized for laboratory confirmed respiratory syncytial virus or influenza virus infection
Source: PLoS One. 2021 Jul 22;16(7):e0253161. doi: 10.1371/journal.pone.0253161 (PMC8297903; doi:10.1371/journal.pone.0253161)
Supplement: Attachment — Submitted filename: PlosOne_SwissRSV_review.docx [file pone.0253161.s003.docx]

I thank the authors for revising their manuscript to make it clearer and transparent. Responses are also well organized inside the Response to Reviewers file. This research shows that it is not easy differentiating adverse clinical outcomes between viral pathogens, especially on viruses that are detected less frequently.

With this revised analysis, the authors could not find any difference in severity between RSV and influenza. However, higher leucocyte counts, and presence of chest infiltrates increased the risk of ICU admission/death. Importantly, RSV patients also had higher median leucocyte counts and number of chest infiltrates. These baseline differences make any inference attributable to the pathogen problematic. More needs to be done in order to understand the relationship between the 2 predictors of interest and RSV/influenza.

It will be useful for the authors to give a plausible explanation for their findings and discuss the role of WBC and chest infiltrates in viral etiology, especially if others have found similar clinical characteristics between RSV and influenza. What is the possible pathway here? Eg: (RSV -> higher leucocyte -> ICU/death **or** RSV -> higher leucocyte -> ICU/death). Discussing this allow the readers to understand if these findings are biologically plausible or due to self-selection factors (ie: RSV patients coming into the ED at a sicker phase of illness).

Minor comments:

1. In Study Setting and Population, “who’s condition required hospital admission.” Should be whose.
2. In Data Collection: week 44 to 16, did you mean epi weeks or calender week? Could just specify.
