# Supplementary material for: Clinical outcomes of adults hospitalized for laboratory confirmed respiratory syncytial virus or influenza virus infection
Source: PLoS One. 2021 Jul 22;16(7):e0253161. doi: 10.1371/journal.pone.0253161 (PMC8297903; doi:10.1371/journal.pone.0253161)
Supplement: Attachment — Submitted filename: Response_to_Reviewers.docx [file pone.0253161.s004.docx]

**Rebuttal No. 2 (PONE-D-21-03134_R1)**

**Clinical outcomes of adults hospitalized for laboratory confirmed respiratory syncytial**

**virus or influenza virus infection.**

**PLOS ONE**

We would like to thank all reviewers again for their efforts and are pleased that our manuscript comes close to meeting the PLOS ONE publication criteria. Thank you for inviting us to submit a revised manuscript. We hope that we were able to adequately address the points raised.

Please find below our response to reviewer #1.

Reviewer #1: Major comment:

With this revised analysis, the authors could not find any difference in severity between RSV and influenza. However, higher leucocyte counts, and presence of chest infiltrates increased the risk of ICU admission/death. Importantly, RSV patients also had higher median leucocyte counts and number of chest infiltrates. These baseline differences make any inference attributable to the pathogen problematic. More needs to be done in order to understand the relationship between the 2 predictors of interest and RSV/influenza.

It will be useful for the authors to give a plausible explanation for their findings and discuss the role of WBC and chest infiltrates in viral etiology, especially if others have found similar clinical characteristics between RSV and influenza. What is the possible pathway here? Eg: (RSV -> higher leucocyte -> ICU/death or RSV -> higher leucocyte -> ICU/death). Discussing this allow the readers to understand if these findings are biologically plausible or due to self-selection factors (ie: RSV patients coming into the ED at a sicker phase of illness).

**Authors’ response:**

We agree that the association between higher leukocyte count/presence of chest infiltrate and ICU admission or death disserves an in-depth discussion. After reconsidering our findings and looking again into the literature, we feel that both considerations remain speculative.

We suggest the following explanation to be added to the discussion section (pages 11-12, line 228-247):

“There was a statistically significantly higher proportion of leukocytosis among patients with RSV compared to those with influenza, albeit with most patients having only mild leukocytosis at most. In multivariable analysis, leukocytosis but not RSV was associated with more severe clinical course. The association between RSV and leukoyctosis has received some attention recently. A study of 243 US adults with RSV showed that 11% had leukocytosis with no difference between those with moderate to severe disease (10%) and those with milder illness (12%, p=0.8). In this study, patients with more severe disease had more frequently pneumonia (Belongia et al. OFID 2018). In a large Chinese study of 1046 adults with RSV, 38% had leukocytosis overall including 44% of those with influenza coinfection while even with these large case numbers the authors were unable to find an association between leukocytosis and severity or radiologic infiltrates (Cui et al. Plos One 2016). In children with RSV, one study found leukocytosis in 24% of those with fever and 18% of those without fever. In those with fever, there was also a higher likelihood of bacterial infection, but among febrile children with a white blood count (WBC) count below 30,000 per milliliter, there was no difference in the WBC between those with and those without bacterial superinfection (Purcell et al. PIDJ 2007). Therefore, it remains speculative, whether the observed higher WBC count in patients with RSV compared to influenza in our study is related to more frequent bacterial superinfections (antibiotic therapy had a non-significantly higher point estimate in RSV than influenza) or to is a marker of more severe clinical presentation of  a viral infection, regardless of whether RSV or influenza. WBC was previously shown to have similar prognostic accuracy as CRP for severe outcome in community acquired pneumonia but without being sensitive or specific enough to work as a predictor on its own (Christ-Crain et al. Crit. Care 10, R96 2006).”

For better transition, we also slightly adjusted the first sentence in the subsequent paragraph (page 12, lines 248-250):

“In contrast to previous results that pointed towards older patients and patients with comorbidities (4, 10), our primary findings of serious outcomes in patients with RSV were independent of age and comorbidities.”

Reviewer #1: Minor comments:

1. In Study Setting and Population, “who’s condition required hospital admission.” Should be whose.

**Reply:** Thank you very much. We have corrected this.

2. In Data Collection: week 44 to 16, did you mean epi weeks or calendar week? Could just specify.

**Reply:** Thank you very much. We considered calendar weeks. We clarified this within the text (page 6, line 104-105):

“This prospective surveillance starts in calendar week 44 in the previous year and extends to calendar week 16 in the following year.”
